# Tuberculosis

**Published:** 1892-12

**Authors:** John M. Parker


### COMMUNICATION.

### TUBERCULOSIS.

To the Editors of the Journal of Comparative Medicine.

*Gentlemen:*—In the September number of the *Veterinary Journal* (Fleming's) I notice an article by Dr. Pearson of the University of Pennsylvania. In speaking of the control of tuberculosis, he says:

"The cure consists, of course, in removing all affected animals and thoroughly disinfecting the stable. Then, if examinations can be made at short intervals, and other cases, if they develop, be removed promptly, the prospects will be quite good for the health of the remaining animals."

I wish to take exception to that last statement. The remaining animals will be every bit as liable to contract the disease as the first lot, and unless you take steps to improve the vitality of the animals, to *increase their disease-resisting powers*, your labor will be useless. We hear and read a great deal about quarantine regulations, and about destroying infected animals, but we don't hear anything about improving the hygienic surroundings of the animals, or breeding for the health and strength of the animals, instead of their milking qualities. The most important factor, from the breeder's point of view (if they consider the matter at all) is, what bull can I use so as to get a big milker. It is not what dam or sire will I use to get a good constitution.

The constitution, *the most important factor in resisting disease*, is lost sight of entirely. Not only that, but the dairy cow (naturally, perhaps, but at the expense of constitution) is fed and milked to its utmost capacity; and, still further, the milking is kept up until the cow is almost ready to drop her calf. If a woman continues to nurse one child up to the time she gives birth to the second, the newcomer, in all probability, will be weak and puny. In the same way your dairy stock must suffer deterioration, if you "burn the candle at both ends," by persisting in raising calves year after year and milking your cows close up to the date of calving. Besides this, many of your dairies are far from what they should be from a sanitary point of view.

How many of your barnyards have their water supply from wells in or near the barnyards? How many of our barns have piles of rotting manure immediately under the barn? And on the other hand, how many have sufficient air and ventilation? You know and I know of barns where, if a lantern is hung up at night time, it will go out for want of air in a short time. A farmer will tell you he has a nice, comfortable, warm barn. These, nice, comfortable, warm barns are responsible, to a great extent, for tuberculosis. How are they heated? not by steam or hot air, but by the exhalations from the animal body. The more overcrowded the barn, the greater the "comfort and warmth" (?)

These are some of the things that are responsible for tuberculosis, and until this state of things is altered, we will have tuberculosis for our guest, in spite of even the killing off of diseased (and sometimes healthy) animals.

Yours truly,

JOHN M. PARKER, D.V.S.

